# T-cell expression of Bruton’s tyrosine kinase promotes autoreactive T-cell activation and exacerbates aplastic anemia

**DOI:** 10.1038/s41423-019-0270-9

**Published:** 2019-08-20

**Authors:** Simo Xia, Xiang Liu, Xuetao Cao, Sheng Xu

**Affiliations:** 1 National Key Laboratory of Medical Immunology, Institute of Immunology, Second Military Medical University, 200433 Shanghai, China; 2 Institute of Immunology, Zhejiang University School of Medicine, 310058 Hangzhou, China; 3 Department of Immunology & Center for Immunotherapy, Institute of Basic Medical Sciences, Peking Union Medical College, Chinese Academy of Medical Sciences, 100005 Beijing, China; 4 College of Life Science, Nankai University, 30071 Tianjin, China

**Keywords:** BTK, aplastic anemia, TCR signaling, bone marrow failure, PLCγ1, Autoimmunity, Cellular immunity

## Abstract

The role of Bruton’s tyrosine kinase (BTK) in BCR signaling is well defined, and BTK is involved in B-cell development, differentiation, and malignancies. However, the expression of Btk in T cells and its role in T-cell function remain largely unknown. Here, we unexpectedly found high expression and activation of BTK in T cells. Deficiencies in BTK resulted in the impaired activation and proliferation of autoreactive T cells and ameliorated bone marrow failure (BMF) in aplastic anemia. Mechanistically, BTK is activated after TCR engagement and then phosphorylates PLCγ1, thus promoting T-cell activation. Treatment with acalabrutinib, a selective BTK inhibitor, decreased T-cell proliferation and ameliorated BMF in mice with aplastic anemia. Our results demonstrate an unexpected role of BTK in optimal T-cell activation and in the pathogenesis of autoimmune aplastic anemia, providing insights into the molecular regulation of T-cell activation and the pathogenesis of T-cell-mediated autoimmune disease.

## Introduction

Bruton’s tyrosine kinase (BTK) is a nonreceptor protein tyrosine kinase belonging to the Tec family and plays key roles in B-cell development and BCR signaling.^1^ Its mutations lead to X-linked agammaglobulinemia, which is characterized by a lack of B lymphocytes and the absence of all classes of immunoglobulins, in humans and mice.^2,3^ Further studies have revealed a key role for BTK in BCR and the pre-BCR signaling pathway that regulates B-cell development, proliferation, survival, and function.^1,4^ Evidence has also shown that BTK is strongly expressed in many types of B-cell leukemia and lymphoma and is key to tumor cell survival and proliferation.^1^ Inhibitors of BTK have shown prominent antitumor activity in patients with various B-cell malignancies.^1^ Recently, ibrutinib, a small molecule inhibitor of BTK, was approved by the FDA for the treatment of chronic lymphocytic leukemia and mantle cell lymphoma.^5^

Because a major phenotype of impaired B-cell development and function is Btk deficiency, much of the interest in Btk has centered around B cells. However, BTK is expressed in many hematopoietic cells, and several recent studies have suggested a more general role for Btk in immune regulation and function. BTK expression in myeloid cells is equivalent to that in B cells and is involved in Toll-like receptor (TLR) signaling by binding to TLRs and phosphorylating Mal.^6,7^ We previously reported that it can bind MyD88 and TRIF adapters in macrophages.^8^ BTK also participates in antiviral innate immunity and inflammasome activation in myeloid cells.^9,10^ In neutrophils, BTK is required for integrin activation events involved in neutrophil recruitment during sterile inflammation.^11^ BTK is also required for natural killer (NK) cell activation and osteoclast differentiation.^12,13^ Recently, we also revealed the involvement of BTK in the translocation of IFNγ-R2 from the Golgi to the membrane.^14^ However, the role of BTK in T cells has not been elucidated, probably due to the paucity of transcription in T cells.^15,16^

Here, we show that the BTK protein is indeed expressed at a certain level in T cells, especially in memory-phenotype T cells. Btk-deficient CD4^+^ T cells exhibited impaired proliferation and differentiation and ameliorated bone marrow failure (BMF) in AA mice after adoptive transfer. After TCR engagement, BTK was activated and subsequently activated the proximal TCR signal molecule PLCγ1, which amplifies downstream TCR signals and facilitates T-cell activation and expansion. The administration of the BTK inhibitor acalabrutinib reduced T-cell responses in AA mice and suppressed BMF. Our study provides evidence for an expanded role of BTK in T cells for optimal TCR-dependent signaling and reveals the role of BTK in the pathology of T-cell-mediated immune diseases.

## Results

### BTK expression and activation in T cells

**Fig. 1:**
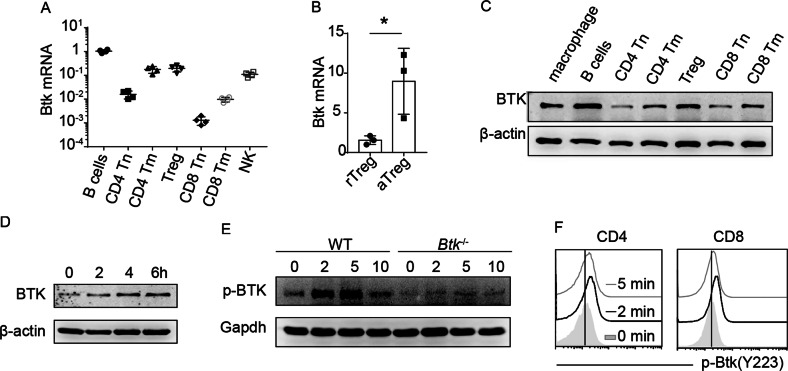
Btk expression and activation in T cells. **a** The expression of Btk mRNA normalized to that of actin in different subsets of lymphocytes. **b** The expression of Btk in rTreg and aTreg cells. **c** Western blot analysis of BTK protein levels in different cell subsets. **d** Immunoblot of BTK expression after TCR engagement. **e** Immunoblot of p-BTK (Y223) in WT and Btk^−/−^ CD4^+^ T cells at different times after TCR engagement. **f** Phosflow analysis of p-BTK (Y223) after TCR engagement. The data shown are the means ± SD and are representative of at least three independent experiments with similar results. *P < 0.05. Tn, naïve T cells; Tm, effector/memory T cells; NK, natural killer cells

To examine whether Btk functions in T cells, we first detected Btk expression in different T-cell subsets. To eliminate B-cell contamination, these T-cell subsets were sorted twice by flow cytometry to guarantee a purity >99%. The mRNA level in naïve CD4^+^ and CD8^+^ T cells was much lower than that in B cells (Fig. 1a); it was only 0.1–1% of that in B cells, which is consistent with previous reports.^15^ The expression of Btk in CD4^+^ T cells was ~10-fold higher than that in CD8^+^ T cells, which was still only ~1% of that in B cells. Furthermore, Btk mRNA was upregulated ~10-fold in effector/memory-phenotype T cells to a level ~1–10% of that in B cells. We also observed high expression of Btk mRNA in Treg cells. Considering that Treg cells can be subdivided into activated Treg (aTreg) cells and resting Treg (rTreg) cells,^17^ we isolated these subsets and found that Btk expression was low in rTreg cells but high in aTreg cells (Fig. 1b), which are CD44^hi^CD62L^low^ and are similar to effector/memory-phenotype T cells.

Surprisingly, the protein level of BTK in T cells, especially in effector/memory T cells, was detectable and comparable to that in B cells (Fig. 1c). IL2-inducible T-cell kinase (ITK) is the analog of BTK in T cells and thus shares some similarity with BTK. To exclude the nonspecific recognition of ITK by the anti-BTK antibody, we overexpressed BTK and ITK in HEK293 cells, and we found that the BTK antibody did not react with ITK (data not shown). Furthermore, the BTK antibody detected obvious BTK expression in WT CD4^+^ and CD8^+^ T cells, but not in Btk^−/−^ T cells (Supplementary Fig. 1A), validating the specificity of the antibody. In addition, BTK expression increased along with the activation of T cells (Fig. 1d). Furthermore, TCR engagement activated BTK kinase in WT T cells but not in Btk^−/−^ T cells (Fig. 1e), which was confirmed by intracellular Phosflow analysis (Fig. 1f). All these data strongly suggest a role for Btk in T-cell responses.

### T-cell expression of BTK exacerbates T-cell-mediated bone marrow failure

**Fig. 2:**
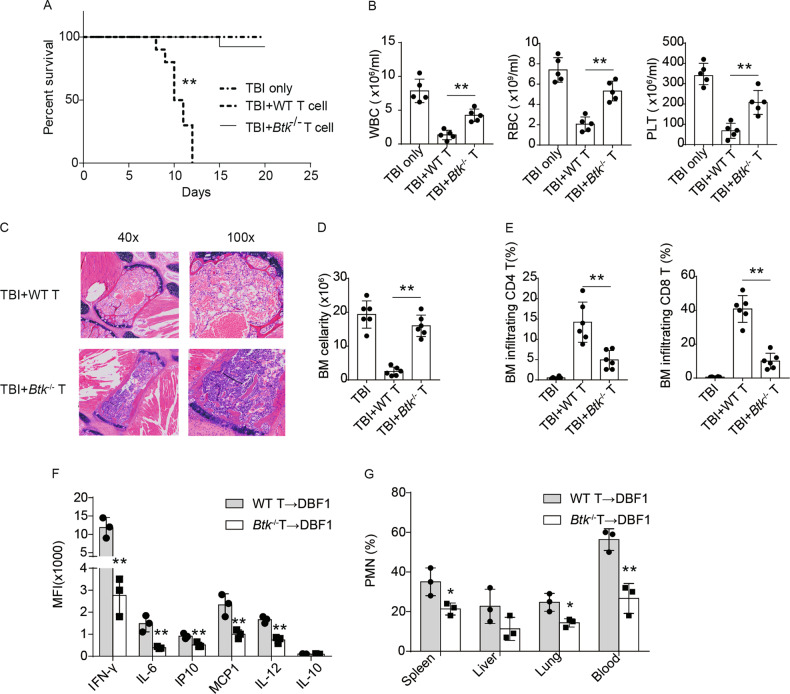
Induction of ameliorated BMF by Btk-deficient T cells. **a** Kaplan–Meier survival estimates for aplastic anemia model mice that received WT or Btk^−/−^ T cells. **b** The number of WBCs, RBCs, and platelets (PLTs) in peripheral blood from AA mice. **c** Representative H&E staining of sternum bone marrow from AA mice; left, 40×; right, 100×. **d**, **e** The BM cellularity and BM infiltration of CD4^+^ T and CD8^+^ T cells were assessed. **f** Cytokine production in the serum was assessed with the BCA assay. **g** The percentages of PMN in the peripheral blood and organs were determined by flow cytometry. The data shown are the means ± SD and are representative of at least three independent experiments with similar results. *P < 0.05; **P < 0.01

As Btk was activated during T-cell activation, we sought to determine whether optimal T-cell responses and T-cell function require Btk expression. To this end, we transferred donor pan T cells derived from WT mice and Btk^−/−^ mice into sublethally irradiated BDF1 recipients to establish an immune-mediated aplastic anemia (AA) model.^18,19^ Graft-vs-host responses cause severe BM destruction and blood pancytopenia in the host, which is mainly attributed to T-cell responses.^20,21^ T-cell development was normal in Btk^−/−^ mice^3^ (Supplementary Fig. 2). As expected, mice that received WT littermate T cells all died within 12 days, and they had a median survival time of 10.5 days. However, most mice that received Btk^−/−^ T cells survived for more than 20 days (Fig. 2a). We further examined cytopenia in the blood. The transfer of Btk^−/−^ T cells, compared with that of WT T cells, caused significantly less severe pancytopenia in the blood (Fig. 2b). These data suggest that Btk contributes to the pathology of immune-mediated AA.

Next, we examined BM hematopoiesis. Less severe BM destruction (Fig. 2c) and relatively normal BM cellularity (Fig. 2d) were found in recipients that received Btk^−/−^ T cells. These recipients also had fewer infiltrating CD4^+^ and CD8^+^ T cells in the BM (Fig. 2e), indicating reduced immune responses and destruction of the BM. In addition, the levels of inflammatory cytokines such as IFN-γ, IL-6, MCP1, and IL-12p70 were significantly reduced in transferred Btk^−/−^ T cells (Fig. 2f), which is consistent with a reduction in neutrophil infiltration in the blood, liver, lung, and spleen (Fig. 2g). The reduced inflammation was not caused by increased inhibitory cytokines, as IL-10 levels were normal in these mice. Thus, all of the data above suggest a relatively reduced inflammatory response and immune destruction of the BM in these Btk^−/−^ T-cell-reconstituted mice.

A previous microarray of T cells from AA patients (GSE3807) showed increased Btk mRNA in peripheral T cells.^22^ We also sorted T cells from a mouse AA model and found that the level of the BTK protein was slightly increased (Supplementary Fig. 1B, C). Altogether, these data support the previously unknown role of BTK kinase in immune-mediated BM failure and AA.

### BTK expression in T-cells exacerbates acute GVHD

**Fig. 3:**
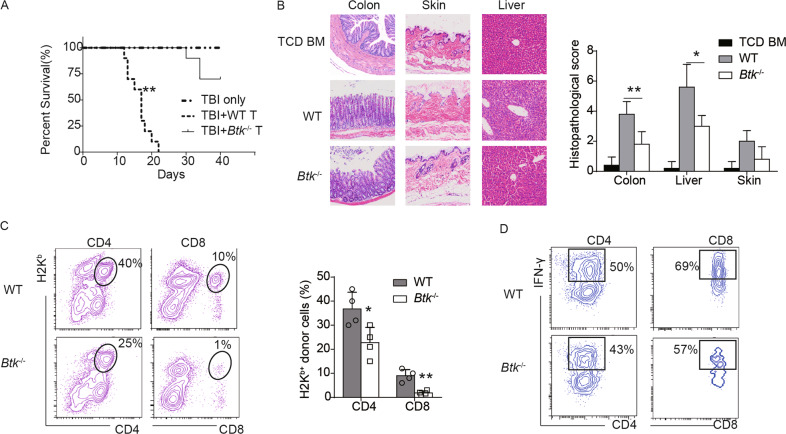
Induction of moderate GVHD by Btk-deficient T cells. **a** Kaplan–Meier survival estimates for aGVHD mice that received TCD-BM plus WT or Btk^−/−^ T cells. **b** Representative H&E staining (left) and pathological scores (right) of the colons, skin and livers of the aGVHD mice in **a**; 100×. **c** Representative flow cytometry plots (left) and the percentages (right) of donor-derived T cells (H2K^b+^) in the spleens of aGVHD mice that received WT or Btk^−/−^ T cells. **d** Representative flow cytometry plots of IFN-γ-secreting T cells in the spleen. The cells were gated on H2K^b+^CD4^+^ or H2K^b+^CD8^+^ T cells. The data shown are the means ± SD and are representative of at least three independent experiments with similar results. *P < 0.05; **P < 0.01

To further confirm the role of BTK in T-cell responses and T-cell pathology, we referred to the MHC-mismatched acute GVHD model. Lethally irradiated Balb/C mice were transplanted with T-cell-depleted bone marrow from B6 mice with or without T cells from WT or Btk^−/−^ mice. As expected, mice that received WT T cells died of GVHD around day 17, and they all died by day 20 (Fig. 3a). However, Balb/C mice that received Btk^−/−^ T cells presented only mild disease, and most of them survived for more than 40 days posttransplantation (Fig. 3a). Histological examination showed an obvious reduction in inflammation in the GVHD target organs (skin, intestine, and liver) (Fig. 3b). These data strongly suggest that the inactivation of Btk signaling in T cells prevents acute GVHD in recipient mice.

To elucidate the influence of Btk deprivation on T-cell function, we further examined the T-cell responses in acute GVHD. Compared with those that received WT T cells, mice that received with Btk^−/−^ T cells showed a reduced number and lower percentage of H2K^b+^ donor CD4^+^ and CD8^+^ T cells (Fig. 3c). IFN-γ production by donor T cells was also reduced (Fig. 3d). Together, these data confirm that Btk deficiency impairs T-cell responses and alleviates acute GVHD.

### BTK promotes allogeneic T-cell proliferation

**Fig. 4:**
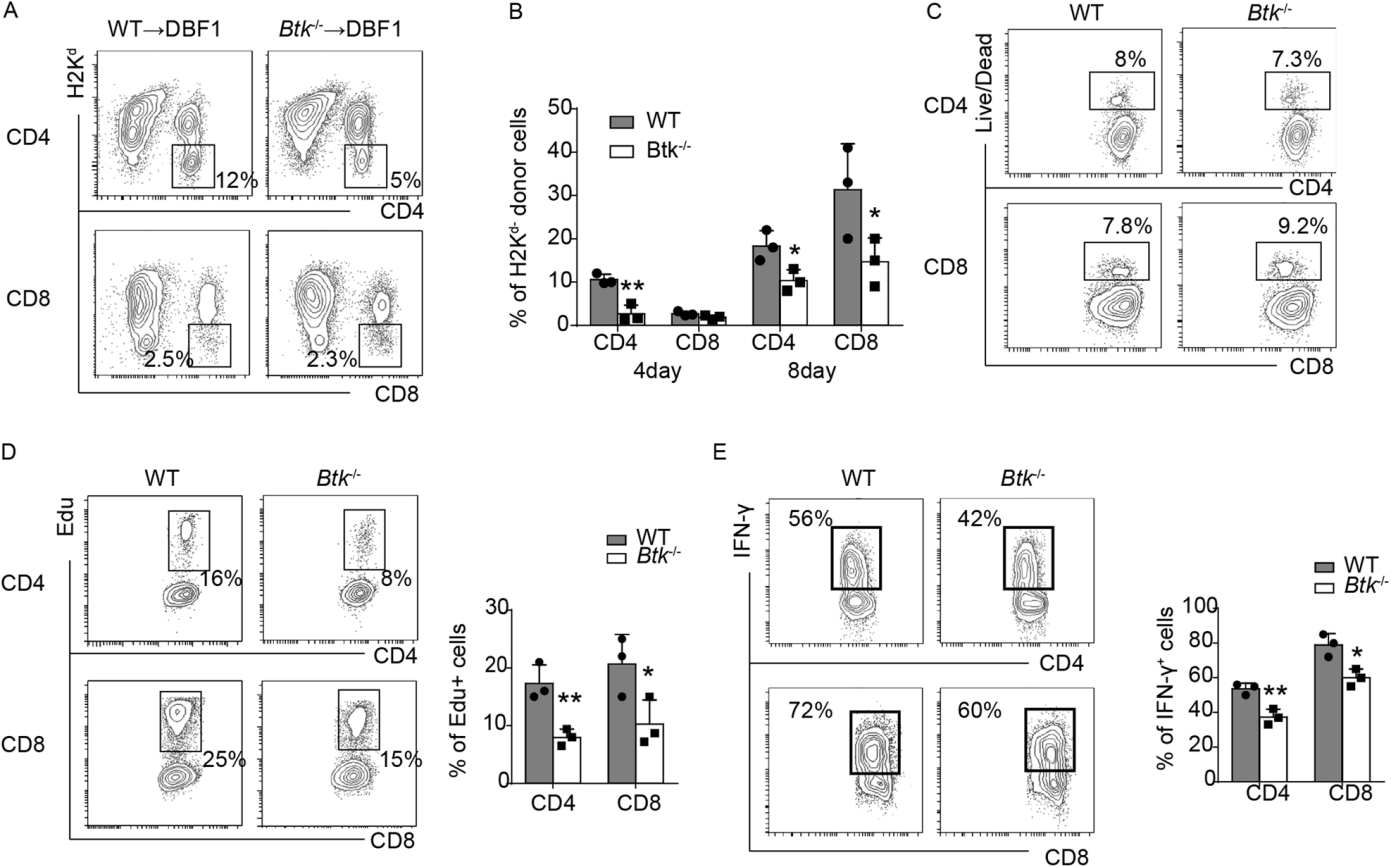
Reduction in T-cell proliferation by Btk deficiency in aplastic anemia. **a** Representative flow cytometry plots of donor CD4^+^ and CD8^+^ T cells (H2K^d−^) from the spleens of irradiated DBF1 mice that received WT or Btk^−/−^ T cells. **b** The percentages of donor CD4^+^ and CD8^+^ T cells in AA mice at the indicated times after T-cell transfer. **c** Representative flow cytometry plots of dead cells from the spleens of DBF1 AA mice that received WT or Btk^−/−^ T cells. The cells were gated on H2K^d−^CD4^+^ or H2K^d−^CD8^+^ T cells. **d** Representative flow cytometry plots (left) and the percentage (right) of EdU-positive donor T cells (gated on CD4^+^H2K^d−^ or CD8^+^H2K^d−^ T cells) in the spleens of irradiated DBF1 mice that received WT or Btk^−/−^ T cells. **e** Representative flow cytometry plots (left) and the percentages (right) of IFN-γ-secreting T cells from the spleens of irradiated DBF1 mice that received WT or Btk^−/−^ T cells (gated on CD4^+^H2K^d−^ or CD8^+^H2K^d−^ T cells). The data shown are the means ± SD and are representative of at least three independent experiments with similar results. *P < 0.05; **P < 0.01

Upon examining T-cell responses in the AA model, we found a reduced number of H2K^d−^ allogeneic T cells in mice that received Btk^−/−^ T cells (Fig. 4a). In the initial phase, the most obvious reduction was in the number of CD4^+^ T cells, and during the late phase, the number of both CD4^+^ and CD8^+^ T cells was significantly reduced (Fig. 4b), indicating that the decreased number of CD8^+^ cells was caused by reduced CD4^+^ T-cell responses. To determine whether the reduced number of T cells in the spleen was due to defects in T-cell migration or emigration, we measured the number of T cells in the lymph nodes (LNs), blood, and lungs (Supplementary Fig. 3A, B). Btk^−/−^ T cells migrated into peripheral tissues, and a similar decline in the number of Btk^−/−^ donor T cells was observed in these tissues, suggesting that Btk^−/−^ T cells exhibited normal migration ability. CXCR4 is a bone homing receptor that is responsible for T-cell migration to the BM in the AA model.^23^ However, no significant difference in CXCR4 expression was found in WT and Btk^−/−^ T cells in the spleen (Supplementary Fig. 3C, D). In addition, Btk^−/−^ T cells migrated to the BM, although a reduced number were found to have migrated (Supplementary Fig. 3A, B). Bone marrow-infiltrating alloreactive T cells express relatively high levels of CXCR4; however, there was similar CXCR4 expression in WT and Btk^−/−^ T cells (Supplementary Fig. 3C, D). These data suggest that Btk^−/−^ T cells migrate normally into the periphery and suggest that the reduced number of T cells may be the result of defective T-cell responses.

The reduction in CD4^+^ T-cell responses observed in Btk^−/−^ cells was also confirmed in a T-cell-mediated IBD model generated by transferring naïve CD4^+^ T cells into Rag2^−/−^ recipients (Supplementary Fig. 4). Btk deficiency alleviated colitis (Supplementary Fig. 4A–C) and led to impaired CD4^+^ T-cell accumulation and inflammation in the spleen, draining LNs and lamina proper lymphocytes (Supplementary Fig. 4D).

To understand the mechanism by which Btk-deficient T cells fail to generate abundant allogeneic T cells, we analyzed the proliferation and cell death of donor T cells in the AA model. There were no differences in cell death between WT and Btk^−/−^ T cells (Fig. 4c). However, more EdU incorporation was found in H2K^d−^ donor T cells from WT mice than in those from Btk^−/−^ mice (Fig. 4d). Reduced EdU incorporation in Btk^−/−^ CD4^+^ T cells was also confirmed in mice with T-cell-mediated colitis (Supplementary Fig. 4E). Meanwhile, the number of IFN-γ-secreting cells was also slightly reduced in Btk^−/−^ T cells (Fig. 4e). Thus, the decreased number of T cells in DBF1 mice that received Btk^−/−^ cells was caused by reduced T-cell proliferation in response to allogeneic antigen, and hypoproliferation may also lead to reduced effector T-cell generation in Btk^−/−^ T cells.

### Intrinsic Btk is responsible for optimal T-cell proliferation

Next, we aimed to elucidate the mechanisms of reduced cell proliferation of Btk^−/−^ T cells. As Treg cells express high levels of Btk and play important roles in repressing allogeneic T-cell responses, we wondered whether Btk deficiency causes aberrant Treg-cell development, which may lead to restricted T-cell proliferation. However, the generation of Foxp3^+^ Treg cells was consistent between WT and Btk^−/−^ mice both in the thymus and periphery (Supplementary Fig. 5A, B). In addition, after transfer into DBF1 mice, there was also no obvious difference in the Treg-cell population between WT and Btk^−/−^ T cells (Supplementary Fig. 5C). One week after transfer, the number of donor-derived Treg cells was greatly reduced in the recipients. A robust reduction in the number of Treg cells in severe AA patients has also been reported.^24^ These data suggest that BTK may not promote the proliferation and expansion of T cells by inhibiting Treg-cell generation. Furthermore, like WT Treg cells, Btk-deficient Treg cells inhibited naïve T-cell proliferation (Supplementary Fig. 5D, E), ruling out the possibility of an enhancement of the regulatory function of Btk^−/−^ Treg cells.

**Fig. 5:**
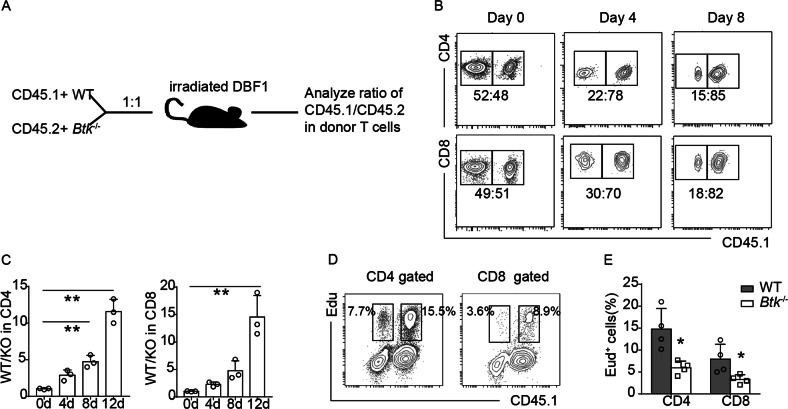
Intrinsic Btk is responsible for defective T-cell proliferation. **a** A schematic of the experimental approach for the analysis of the extrinsic and intrinsic roles of Btk. **b** Analysis of the WT (CD45.1^+^) and Btk^−/−^ (CD45.1^−^) ratio in CD4^+^ and CD8^+^ T cells in the spleen on different days after adoptive transfer into DBF1 mice gated on H2K^d−^ donor T cells. **c** Statistic analysis of the WT:Btk^−/−^ T-cell ratio at different times after transfer. **d**, **e** Representative flow cytometry plot (**d**) and the percentages (**e**) of EdU incorporation in WT or Btk^−/−^ T cells transferred into DBF1 mice at a 1:1 ratio (gated on CD4^+^H2K^d−^ or CD8^+^H2K^d−^) in the spleen. The numbers in the plot are the percentages of total CD45.1^+^ or CD45.1^−^ T cells that are CD45.1^+^ EdU^+^ or CD45.1^−^ EdU^+^. The data shown are the means ± SD and are representative of at least three independent experiments with similar results. *P < 0.05; **P < 0.01

The reduced proliferation of Btk^−/−^ T cells in the Btk^−/−^ AA model may also be caused by reduced levels of inflammatory cytokines (e.g., IL2). To examine this hypothesis, we cotransferred WT (CD45.1^+^CD45.2^−^H2K^d−^) and Btk^−/−^ (CD45.1^−^CD45.2^+^H2K^d−^) T cells at a 1:1 ratio into irradiated DBF1 mice (Fig. 5a). After AA induction, WT T cells gradually dominated the donor T-cell population (Fig. 5b). There were ~10-fold more WT T cells than Btk^−/−^ T cells after 12 days (Fig. 5c). In addition, these Btk^−/−^ T cells also showed reduced EdU incorporation compared with that shown by WT T cells (Fig. 5d, e), suggesting impaired proliferation of these Btk-deficient cells. The reduced proliferation of Btk^−/−^ T cells cannot be attributed to reduced cytokine production, as this would have been rescued by mixed transfer with WT T cells. Thus, all of these data suggest an intrinsic and indispensable role of BTK in T-cell proliferation.

### Btk promotes T-cell activation and expansion

**Fig. 6:**
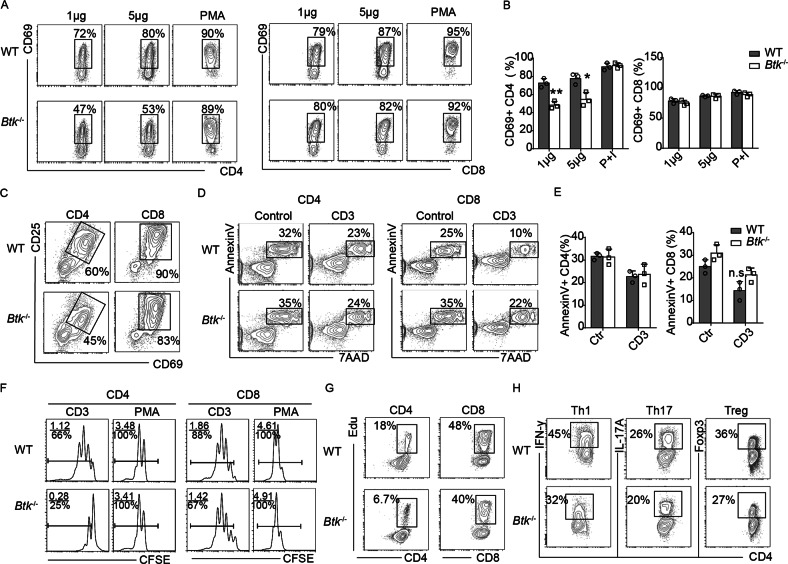
Btk deficiency ameliorates T-cell activation and proliferation. Flow cytometry **a** and analysis **b** of CD69 expression in CD4^+^ and CD8^+^ T cells from WT and Btk^−/−^ mice 4 h post anti-CD3/anti-CD28 or PMA/ionomycin stimulation. **c** Flow cytometry analysis of CD25/CD69 expression 1 day after activation. **d**, **e** Apoptosis of CD4^+^ and CD8^+^ T cells 1 day after anti-CD3/anti-CD28 stimulation in vitro. **f** CFSE dilution assay of WT and Btk^−/−^ T cells simulated with anti-CD3/anti-CD28 or PMA/ionomycin. The numbers shown in the plot are the division index (upper) and the percentage of proliferated cells (bottom). **g** EdU incorporation in WT and Btk^−/−^ T cells stimulated with anti-CD3 plus anti-CD28. **h** Intracellular cytokine production by differentiated Th1, Th17, and Treg cells from WT and Btk^−/−^ CD4^+^ T cells. The data shown are the means ± SD and are representative of at least three independent experiments with similar results. *P < 0.05; **P < 0.01

We then investigated the role of BTK in T-cell activation and proliferation in vitro. After CD3 and CD28 stimulation, Btk deficiency led to the reduced expression of the early activating marker CD69 (Fig. 6a, b). The decrease was especially significant in CD4^+^ T cells but not in CD8^+^ T cells, consistent with the much lower level of Btk expression observed in CD8^+^ T cells (Fig. 1a). CD25 expression was also decreased in Btk^−/−^ T cells after a long culture period (Fig. 6c). However, when cells were stimulated with PMA plus ionomycin, which bypasses proximal TCR signaling, the activation of Btk^−/−^ T cells was comparable to that of WT cells (Fig. 6a, b). Meanwhile, CD4^+^ T-cell apoptosis was also the same between the two groups (Fig. 6d, e). However, cell death was increased in Btk^−/−^ CD8^+^ T cells, although the difference was not statistically significant (Fig. 6d, e).

To investigate whether Btk influences T-cell proliferation, we used CFSE to track cell division. Btk^−/−^ T cells showed a reduced percentage of dividing cells and a lower division index compared with those shown by WT T cells (Fig. 6f). The proliferation defect was more profound in Btk^−/−^ CD4^+^ T cells. Cell cycle analysis revealed less EdU incorporation in Btk^−/−^ T cells (Fig. 6g), representing a reduced percentage of cells in S phase. As expected, PMA plus ionomycin treatment abrogated the differences between Btk^−/−^ and WT T-cell proliferation and expansion (Fig. 6f). Furthermore, Btk deficiency also inhibited Th1, Th17, and Treg-cell generation (Fig. 6h). To determine whether the suppression of Th-cell differentiation was caused by direct Btk deficiency or occurred after decreased proliferation, differently divided cytokine-positive cells were by CFSE dilution (Supplementary Fig. 6). There was a greater number of highly divided cells that were cytokine- or Foxp3-positive, and this effect was reduced or absent in Btk^−/−^ cells. These data suggest that the reduction in Th-cell generation in Btk^−/−^ cells may be attributed to the reduced proliferation of these cells.

Collectively, these data suggest that Btk has a nonredundant role in T-cell activation and proliferation, especially in CD4^+^ T cells, and that this effect can be rescued by stimulation with PMA plus ionomycin.

### Btk promotes TCR signaling by phosphorylating PLCγ1

**Fig. 7:**
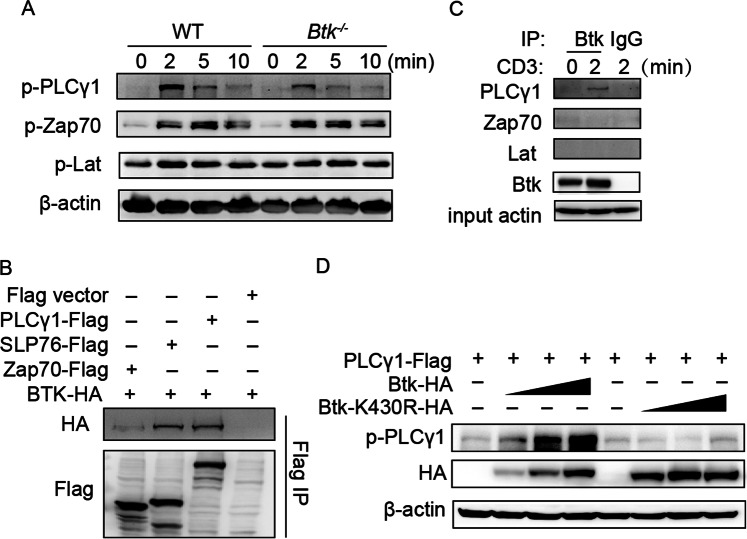
BTK promotes TCR signaling by phosphorylating PLCγ1. **a** WT and Btk^−/−^ CD4^+^ T cells were stimulated with anti-CD3 plus anti-CD28, and the activation of proximal TCR signal molecules was determined by immunoblotting for certain antibodies at the indicated time. **b** BTK-HA was overexpressed in 293T cells with Flag-tagged ZAP70, SLP76 or PLCγ1, and their interaction was determined by immunoblotting after immunoprecipitation with a Flag antibody. **c** CD4^+^ T cells were stimulated with plate-bound anti-CD3 plus CD28, and the cell lysates were immunoprecipitated with an anti-BTK antibody with or without TCR engagement and then analyzed by an immunoblot assay. **d** Immunoblot analysis of PLCγ1 phosphorylation in HEK293T cells transfected with different amounts of BTK or K430R BTK. The data shown are representative of at least three independent experiments with similar results

Considering that Btk deficiency-induced T-cell hyporesponsiveness can be rescued by PMA plus ionomycin stimulation, we sought to determine whether Btk plays a role in proximal TCR signaling. As Btk is a tyrosine kinase, we first examined proximal TCR signal molecule phosphorylation and activation to identify potential targets. There was no difference in Zap70 and LAT phosphorylation in lysates from CD4^+^ T cells but the phosphorylation of PLCγ1 was reduced in Btk^−/−^ cells (Fig. 7a), suggesting that PLCγ1, a critical proximal TCR signal component, may serve as a potential BTK substrate.

To determine whether BTK interacts with PLCγ1 or other proximal TCR molecules, BTK was overexpressed in 293T cells with TCR proximal signal components. In the absence of TCR signaling, BTK was coimmunoprecipitated with PLCγ1 and SLP76 (Fig. 7b). To further evaluate the role of TCR stimulation and the physiological interactions in T cells, Btk was coimmunoprecipitated from naïve or activated CD4^+^ T cells. We found that BTK specifically interacted with PLCγ1 in a TCR stimulation-dependent manner (Fig. 7c), and BTK did not interact with ZAP70, SLP76, or LAT. Furthermore, when coexpressed with PLCγ1, BTK increased PLCγ1 phosphorylation in a dose-dependent manner (Fig. 7d). This function depended on the kinase activity of BTK, as the kinase-inactivated K430R BTK mutation did not phosphorylate PLCγ1. Collectively, these data demonstrate that BTK serves as a positive regulator of proximal TCR signaling and promotes T-cell activation and expansion by targeting PLCγ1 phosphorylation.

### Blocking BTK attenuates T-cell responses and lethal bone marrow failure

**Fig. 8:**
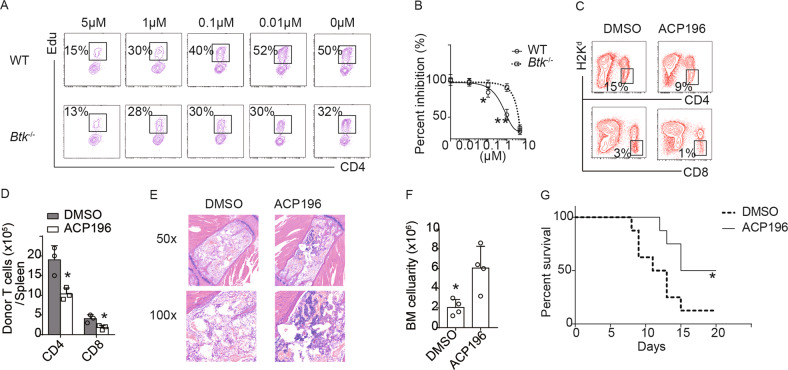
The Btk inhibitor ACP196 ameliorates mouse aplastic anemia by suppressing T-cell activation and proliferation. CD4^+^ T cells were stimulated with anti-CD3 (0.1 μg/ml) plus anti-CD28 after treatment with different concentrations of the selective Btk inhibitor ACP196, and EdU incorporation was determined after 3 days (**a**). The percentage of inhibition is shown in **b**. AA mice were treated with ACP196 or vehicle control, and the infiltration of donor T cells in the spleen was analyzed on day 5 (**c**, **d**). Representative hematoxylin and eosin staining of the BM (**e**, upper 50×; bottom 100×). BM cellularity (**f**) was analyzed on day 10. **g** Kaplan–Meier survival estimates for AA mice treated with ACP196 or vehicle control (*n* = 8). The data represent the mean ± SD and are representative of three independent experiments. *P < 0.05; **P < 0.01

Acalabrutinib (ACP196) is a selective second-generation BTK kinase inhibitor. Importantly, unlike ibrutinib, acalabrutinib rarely inhibits ITK, EGFR, or TEC^25^ and has recently been widely used to ameliorate B-cell-associated BTK activity. Thus, we wondered whether acalabrutinib can also be used therapeutically to inhibit T-cell responses and ameliorate BMF in a mouse AA model. We first investigated the effect of acalabrutinib on T-cell proliferation after stimulation with CD3 and CD28 in vitro. In WT CD4^+^ T cells, 0.1 μM acalabrutinib significantly inhibited T-cell proliferation, as revealed by reduced EdU incorporation (Fig. 8a, b), and the inhibition was more profound at a concentration of 1 μM; however, Btk^−/−^ cells were not inhibited at these concentrations. At a concentration of 5 μM, ACP196 robustly suppressed both WT and Btk^−/−^ T-cell expansion, which may be attributed to off-target effects (Fig. 8a, b). These data confirm an intrinsic and profound role of Btk in CD4^+^ T-cell activation and expansion.

The administration of acalabrutinib from the establishment of the AA model reduced the expansion of donor T cells and diminished the infiltration of T cells in the spleen in vivo (Fig. 8c, d). The BM from acalabrutinib-treated mice showed a significant amelioration of destruction and relatively obvious hematopoiesis (Fig. 8e, f). Notably, half of the mice treated with ACP196 survived for more than 20 days (Fig. 8g); in contrast, control AA mice had a median survival time of 12 days. To test whether acalabrutinib has a therapeutic effect after the onset of disease, we treated mice with ACP196 5 days after T-cell transfer. Acalabrutinib treatment still inhibited the expansion of alloreactive T cells, at least CD4^+^ T cells, in the spleen (Supplementary Fig. 7A, B), and slightly ameliorated bone marrow destruction (Supplementary Fig. 7C). However, mouse survival was not significantly improved after acalabrutinib treatment (Supplementary Fig. 7D). These data indicate that BTK inhibitors can inhibit T-cell responses and thus ameliorate the severity of AA in the early phase and are less effective after the onset of the disease when pathogenic effector T cells are generated. Together, these data suggest that BTK may be involved in T-cell activation and the pathogenesis of acquired AA.

## Discussion

Severe AA is a fatal disease characterized by acquired bone marrow failure and blood pancytopenia.^21,26,27^ Clinical and laboratory evidence suggests that AA is an immune-mediated disease characterized by the active destruction of hematopoietic stem and progenitor cells by T cells in the bone marrow.^21,27,28^ How tolerance is broken and the exact autoantigens are not known, and there are no precise animal models of AA. However, immune-mediated mouse models have been successfully established by transferring parental splenocytes into MHC-mismatched offspring mice.^19^ This model exhibits most clinical features of AA, including BM destruction, HSC deletion, and pancytopenia in the blood. Thus, it can be used to study the mechanism of AA and the efficacy of new therapeutic drugs. Previous studies using this model have demonstrated that Notch and Ezh2 contribute to BMF.^29,30^ We slightly modified the model by depleting non-T cells in the transferred parental splenocytes to exclude the influence of other immune cells, especially B cells and myeloid cells. By using this model, we found that Btk deficiency in adoptively transferred T cells induced moderate BMF in recipient AA mice, which was further demonstrated to be attributed to intrinsic defects in T-cell activation and proliferation.

BTK belongs to the Tec family kinases, which consists of five members: Tec, Btk, Itk, Rlk, and Bmx.^31^ These kinases are unique in that they are the only tyrosine kinases to possess a pleckstrin homology (PH) domain that binds to PtdIns(3,4,5)P3 (PIP3) in the cell membrane. BTK is broadly expressed in hematopoietic cells and has been reported to be involved in the function of B cells,^1^ macrophages,^6,7,9^ neutrophils,^11^ osteoclasts^13^ and NK cells,^12^ mast cells,^32^ etc. No report has declared a role for BTK in T cells until now.

Previously, a low level of Btk mRNA transcription was identified in T cells.^15,16^ Thus, some researchers declared that BTK is not expressed in T cells,^31^ which may have discounted a role for BTK in T-cell activity. Later, its analog ITK was identified. ITK expression is largely limited to T cells and is crucial for T-lymphocyte development and proliferation.^33^ The dogma is that BTK and ITK are responsible for the phosphorylation and activation of downstream effectors in the BCR and TCR signaling pathways,^34^ respectively. In resting lymphocytes, BTK and ITK reside in the cytosol. Upon antigen receptor activation, they are recruited to the plasma membrane through their PH domain. Then, BTK and ITK are activated by Src kinases and proceed to phosphorylate the lipase PLCγ2/ PLCγ1, which cleaves PIP2 in the plasma membrane and generates the secondary messengers IP3 and DAG.^31,35^ The strong homology between Btk and Itk and their apparently similar modes of activation following antigen receptor engagement in B and T cells, respectively, have suggested that they represent division of labor in the two lymphocyte lineages. These findings may further impede the exploration of the role of BTK in T cells.

However, in our study, the levels of Btk mRNA transcription were low, which is consistent with previous reports. The BTK protein was expressed at a level comparable to that in B cells, especially in memory-phenotype T cells. Differences between mRNA and protein expression have been reported.^36,37^ The poor correlation between the levels of mRNA and the levels of protein may be attributed to complicated posttranslational mechanisms and/or different half-lives of the protein in different cells. Furthermore, after TCR engagement, BTK was phosphorylated and activated, strongly indicating a role of BTK in T-cell function. Btk was also demonstrated to be upregulated in peripheral blood T cells in severe AA patients.^22^

After transfer into DBF1 mice, Btk^−/−^ T cells showed relatively less expansion, which resulted in a reduced number of pathological donor T cells and moderate BMF in these mice. A proliferation defect in vivo was first observed in Btk^−/−^ CD4^+^ Th1 cells within 5 days and sequentially and less obviously in CD8^+^ T cells. Furthermore, within 4 h after CD3/CD28 stimulation in vitro, Btk deficiency reduced CD4^+^ T-cell activation but had no influence on CD8^+^ T cells, suggesting that impaired CTL responses may be attributable to defects in CD4^+^ T cells. The Rag2^−/−^ mouse model of T-cell-mediated colitis also revealed a nonredundant role of BTK in CD4^+^ T-cell responses.

The suppressed of T-cell responses in Btk^−/−^ CD4^+^ T cells may be caused by the increased suppression by inhibitory Treg cells. As BTK was highly expressed in Treg cells at both the mRNA and protein levels, we considered whether Btk deficiency might promote Treg development. However, natural Treg development was not impaired in Btk^−/−^ mice. The development of Tregs was also the same in WT and Btk^−/−^ T cells after they were transferred into irradiated DBF1 mice. In addition, WT and Btk^−/−^ Treg cells suppressed T-cell responses to the same extent. These data exclude the possibility that BTK suppressed T-cell responses by regulating Treg-cell generation and function.

Inappropriate apoptosis may underlie the pathogenesis of autoimmune diseases as well as AA. Previous reports have uncovered proapoptotic and antiapoptotic properties of Btk,^38,39^ indicating radically different roles depending on cellular context and stimuli. We did not observe differences in apoptosis between WT and Btk^−/−^ T cells in AA mice in vivo. However, although not statistically significant, apoptosis of CD8^+^ T cells was slightly higher in Btk^−/−^ T cells in vitro, which is consistent with a slightly reduced number of CD8^+^ T cells with a central memory phenotype (CD44^hi^CD62L^+^) in Btk^−/−^ mice (Supplementary Fig. 2). However, the reduction in the number of CD8^+^ T cells with a memory phenotype is not responsible for the reduced T-cell response in the Btk^−/−^ AA model. CD4^+^ T cells are key for mediating BMF and AA.^21,29,40^ In addition, soon after T-cell transfer, there was no difference between WT and Btk^−/−^ CD8^+^ T-cell proliferation, while the proliferation of Btk^−/−^ CD4^+^ T was lower than that of WT cells. Recently, one study reported that the inhibition of BTK by acalabrutinib does not influence activation-induced cell death in human T cells.^41^ Whether BTK also plays a role in T-cell apoptosis remains to be elucidated.

However, the impaired T-cell activation and proliferation in Btk^−/−^ T cells after anti-CD3/anti-CD28 stimulation was abrogated upon treatment with PMA plus ionomycin, suggesting a role for Btk in proximal TCR signaling. Itk has been suggested to have a similar function in T cells as that of Btk in B cells. However, the absence of Itk did not fully abrogate TCR signaling, and Itk has been suggested to be mainly involved in Th2, Th9, and Th17 differentiation^33,42^ rather than in TCR signaling, suggesting that other TEC kinases may also be involved in proximal TCR signaling. For example, Rlk has been indicated to work synergically with Itk,^43^ but Rlk does not possess a PH domain and may not be recruited to the membrane. In our study, we first reported that BTK can function together with ITK in T cells to activate PLCγ1 and amplify TCR signaling.

The Btk kinase inhibitor ibrutinib has been approved by the FDA for clinical therapy for several B-cell lymphomas due to its inhibitory activity of tumor cell survival and proliferation. However, ibrutinib is also able to alleviate the clinical manifestations of acute GVHD^44^ and T-cell-mediated chronic GVHD,^45^ suggesting that an inhibitory effect of ibrutinib in vivo on T cells contributes to a reduction in T-cell pathogenesis. The authors attributed the effect to the direct suppression of ITK in T cells and its indirect suppression in other BTK-expressing cells, such as APCs. Our study suggests that BTK inhibition in T cells should not be ignored and may also be responsible for reduced T-cell pathogenesis after ibrutinib administration.

Recently, ibrutinib has been suggested to have antitumor effects in solid tumors^46^ through the inhibition of BTK in tumor cells or cells in the tumor microenvironment, such as MDSC, monocytes, macrophages and mast cells.^46,47^ In addition, the suppression of ITK may drive Th1 selective pressure in T cells^48^ and thus may also account for the antitumor effect of ibrutinib. However, there are no reports concerning the direct role of Btk in T cells. Our study also serves as a reminder that the effect of BTK in T cells should also be taken into account when using BTK inhibitors for tumor therapy.

In summary, we unexpectedly found that BTK is expressed in T cells and that it is also upregulated in T cells from an AA model and patients with severe AA. After TCR engagement, BTK is activated and contributes to the phosphorylation of PLCγ1, which then exacerbates TCR downstream signals. A deficiency in Btk impairs T-cell activation and proliferation, resulting in ameliorated BMF. In addition, treatment with a selective BTK inhibitor also suppresses AA. Thus, our study revealed an unexpected role of Btk in TCR signaling and in the pathogenesis of acquired AA and possibly other autoimmune diseases.

## Materials and methods

### Mice

C57BL/6 (B6, H-2^b^) and DBA/2 (D2, H-2^d^) mice were purchased from Sipper BK Experimental Animals (Shanghai, China) and crossed to generate B6 × D2 F1 (BDF1, H-2^b/d^) mice. Btk-deficient mice (002536) were originally derived from the Jackson Laboratory and backcrossed to the B6 background (>8 generations), and littermate control mice were used as control WT mice. Age- and sex-matched CD45.1 WT mice were used as controls. The experimental protocols were approved by the Second Military Medical University Committee on Use and Care of Animals.

### Induction of AA

A mouse model of AA was induced as described previously with slight modifications.^19^ Briefly, BDF1 mice were conditioned with 5.5 Gy of total body irradiation (TBI). Pan T cells were isolated by magnetic cell sorting (MACS) from splenocytes from littermate WT or Btk^−/−^ mice, and 5 × 10^6^ pan T cells/mouse were transferred i.v. 6 h after irradiation. The mice were monitored daily for signs of disease and euthanized at the indicated times. Peripheral blood was collected from the heart or the lateral tail vein for complete blood counts or ELISA. Sterna were collected for histology. Lymphocytes were isolated from the spleen or LNs, and BM cells were isolated from bilateral tibias and femurs. For ACP196 treatment of AA, mice were administered vehicle or ACP196 via oral gavage from day 0 after T-cell transfer until sacrifice. In the AA therapy study, mice were administered vehicle or ACP196 from day 5 after T-cell transfer until sacrifice.

### Induction of GVHD

BALB/c recipients (8–10 weeks old) were conditioned with 8 Gy TBI. Within 24 h, the mice were transplanted with donor B6 T-cell-depleted (TCD) BM cells (5 × 10^6^) alone or with T cells (5 × 10^6^) from littermate WT or Btk^−/−^ mice. GVHD severity was assessed by histopathological analysis.^49^

### Histologic examination

Histologic examinations were performed on paraffin-embedded sections that were fixed with 10% formalin and stained with H&E.

### Real-time PCR

Total RNA was extracted from the indicated CD4^+^ T-cell subsets using TRIzol (Invitrogen Life Technologies). cDNA was quantified by quantitative real-time PCR. Real-time PCR was performed with SYBR Green PCR mix on an ABI QuantStudio 7 Flex Real-time PCR system (Applied Biosystems, CA, USA). Transcript abundance was calculated using the 2^−ΔΔCt^ method (normalization to β-actin). The primer sequences were obtained from PrimerBank (https://pga.mgh.harvard.edu/primerbank).

### Immunoprecipitation and western blot analysis

Cells were lysed with cell lysis buffer (CST) supplemented with protease inhibitor cocktail (Calbiochem). The protein concentrations of the extracts were measured with a BCA assay (Pierce). The immunoprecipitation assays and immunoblot assays were performed as previously described.^8^ The blots were incubated with anti-phospho-Btk (Tyr223) (5082; CST), anti-Btk (3532; 8547; CST), anti-Btk (sc28387; Santa Cruz), anti-PLCγ1 Y183 (14008; CST), p-Zap70 Y319 (271; CST), p-Lat Y191 (3584; CST), anti-Zap70 (2705; CST), anti-LAT (9166; CST), anti-SLP76 (4958; CST), anti-PLCγ1 (05–163; Millipore), anti-V5 (13202;CST), or anti-actin (ab3280; Abcam) antibodies and subsequently incubated with HRP-conjugated anti-rabbit or anti-mouse IgG (CST) in TBS containing 5% nonfat dry milk and 0.05% Tween 20. The final reaction was developed with a chemiluminescent system (Pierce).

### Cytokine detection

The serum was collected from the mice, and the levels of the cytokines IFN-γ, IL-6, IP-10, MCP1, IL-12 IL-10, etc. were detected by a cytometric bead array assay according to the manufacturer’s instrument (BioLegend).

### T-cell isolation and in vitro differentiation assays

Spleens were isolated and manipulated through a 40-µm filter, and erythrocytes were lysed with ACK lysis buffer. Different cell subsets were sorted by a fluorescence activated cell sorter (FACS) (Sony S800) according to the following cell markers: naïve CD4 (CD4^+^CD25^−^CD62L^+^CD44^−^); memory CD4 (CD4^+^CD25^−^CD62L^−^CD44^+^); Treg (CD4^+^CD25^hi^); aTreg (CD4^+^CD25^hi^CD44^+^CD62L^−^); rTreg (CD4^+^CD25^hi^CD44^−^CD62L^+^); naïve CD8 (CD8^+^CD44^−^CD62L^−^); memory CD8 (CD8^+^CD44^+^); NK (Nkp46^+^); B cell (B220^+^); and macrophage (F4/80^+^CD11b^+^). Pan T cells were separated by negative selection using a Pan T Cell Isolation kit (Miltenyi Biotec, Germany). CD4^+^ and CD8^+^ T cells were isolated using anti-mouse CD4 and CD8 magnetic particles and separated by MACS.

For in vitro studies, cells were plated at a density of 5 × 10^5^ cells/well in 96-well plates precoated with anti-CD3 (Clone 500A2; BD Pharmingen) and anti-CD28 (clone 37.51; BD Pharmingen) (0.5 μg/ml) or stimulated with a cell stimulation cocktail (eBioscience). To polarize T cells toward a Th1 phenotype, 1 ng/ml mIL-12 (R&D) and 10 μg/ml anti–mIL-4 (BioLegend) were added to culture medium at the time of plating. For Th17 cells, 5 ng/ml mTGF-β, 20 ng/ml mIl-6, 2 ng/ml mIL-23 (Peprotech), 10 μg/ml anti-IFN-γ, and 10 μg/ml anti-IL-4 were added. For Treg induction, 5 ng/ml TGF-β, 100 IU rhIL-2, and 10 μg/ml anti-IFN-γ were added.

### Antibodies and flow cytometry analysis

The antibody used for immunofluorescence staining (anti-phospho-BTK T223 (clone A16128B; BioLegend)) was purchased from eBioscience and BioLegend. Flow cytometry analyses were performed using FACSVantage (Becton Dickinson) as previously described.^50^ The data were analyzed by Flowjo or FACS Diva. For intracellular cytokine staining, cells were restimulated in vitro with a cell stimulation cocktail (plus protein transport inhibitors) (eBioscience) for 6 h.

### Cell proliferation and EdU assays

For the cell proliferation assay, T cells were labeled with 5 μM CellTrace CFSE (Life Technologies) for 15 min at 37 °C, washed with cold medium, and subjected to anti-CD3/anti-CD28 or PMA/ionomycin stimulation. Cell proliferation was measured by CFSE dilution 3 days later. For the EdU incorporation assay, cells were cultured with 20 μM EdU for 8 h (ex vivo assay) or overnight (in vitro assay), and EdU incorporation was detected by flow cytometry according to the manufacturer’s instructions.

### Statistical analysis

The results are expressed as the mean ± SD; unpaired, two-tailed Student’s *t* test (Prism 7; GraphPad Software) was used for statistical comparisons of two groups, and one-way ANOVA followed by Tukey’s multiple comparisons post test was used for comparisons of several groups. Effects on survival were determined using Kaplan–Meier estimates with an applied log-rank test. *P*-values of ≤0.05 were considered significant.

## Supplementary information

Supplementary material
