# Supplementary material for: T-cell expression of Bruton’s tyrosine kinase promotes autoreactive T-cell activation and exacerbates aplastic anemia
Source: Cell Mol Immunol. 2019 Aug 20;17(10):1042–52. doi: 10.1038/s41423-019-0270-9 (PMC7608443; doi:10.1038/s41423-019-0270-9)
Supplement: Supplementary file 1 — Supplementary material [file 41423_2019_270_MOESM1_ESM.doc]

### Supplementary materials for

# T cell expression of Brutons tyrosine kinase promotes autoreactive T cell activation and exacerbates aplastic anemia


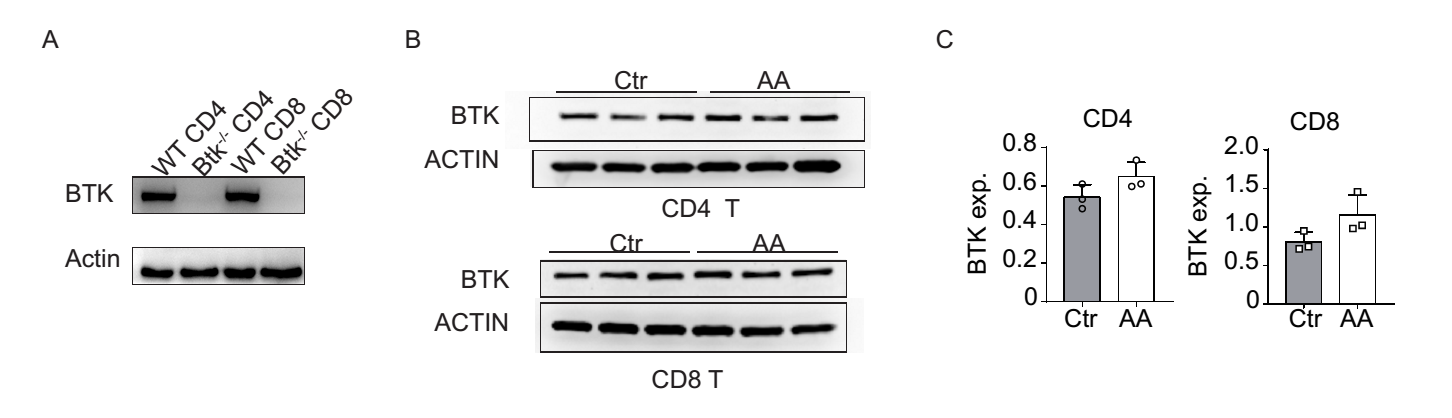


**Supplementary Figure 1. BTK expression in T cells and aplastic anemia models**

(**A**) BTK expression in CD4^+^ and CD8^+^ T cells from WT and Btk^-/-^ mice. (**B**) CD4^+^ and CD8^+^ T cells were isolated from AA mice on day 7, and BTK expression was determined by Western Blot assay; (**C**) Statistic analsis of data in (A). Data represent the mean ± SD or representative from 3 independent experiments.


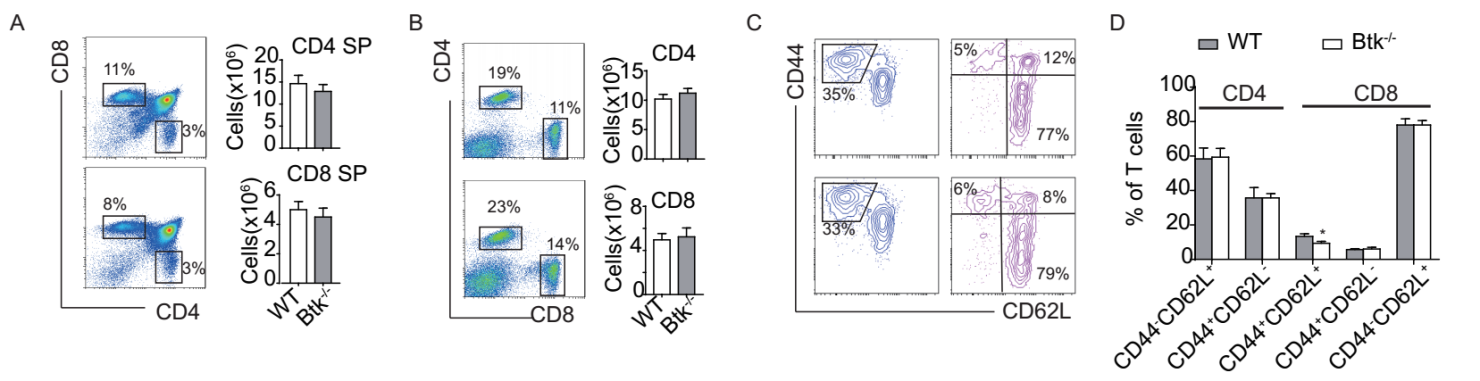
 **Supplementary Figure 2. T cell development in Btk deficient mice**

(**A**)Representative flow cytometry plot shown T cell develompent in the thymus, absolute number of CD4 and CD8 single positive (SP) cells were shown on the right; (**B**) Representative plot shown periphery mature T cells in the spleen, absolute number of CD4 and CD8 T cells were shown on the right; (**C**) Representative plots shown different subsets of CD4 and CD8 T cells in littermate WT and Btk^-/-^ mice. (**D**) Different T cell subsets in WT and littermate Btk^-/-^ mice. Data represent the mean ± SD or representative from 3 independent experiments. * P<0.05


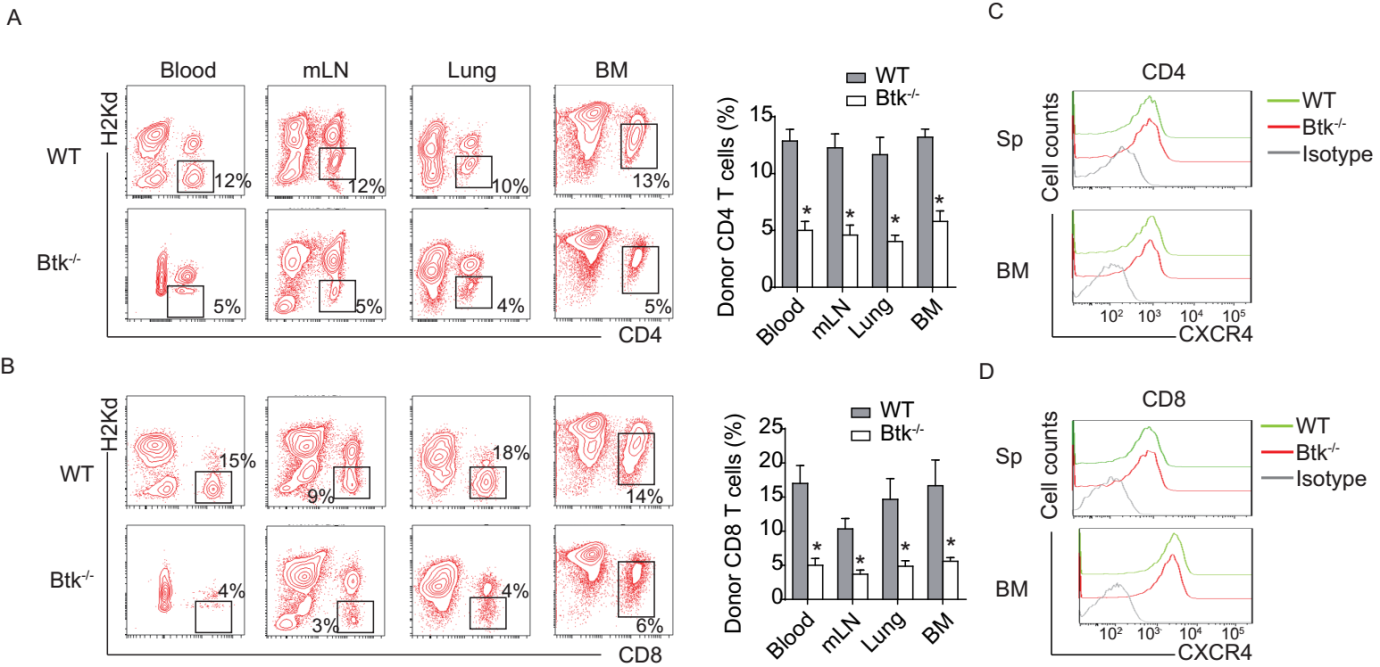


**Supplementary Figure 3. BTK deficiency suppresses T cell response in aplastic anemia without impairing T cell migration**

(**A&B**) Representative flow cytometry plot shown percent of donor derived CD4^+^ T (A) and CD8^+^ T (B) cells in the blood, mLN, lung 5 days post T cell transfer, and in the BM 8 days post tranfer. Statistical analysis were shown on the right; (**C&D**) Representative plot shown CXCR4 expression in donor CD4^+^ (C) and CD8^+^ (D) T cells in the spleen and BM. Data represent the mean ± SD or representative from 3 independent experiments. * P<0.01


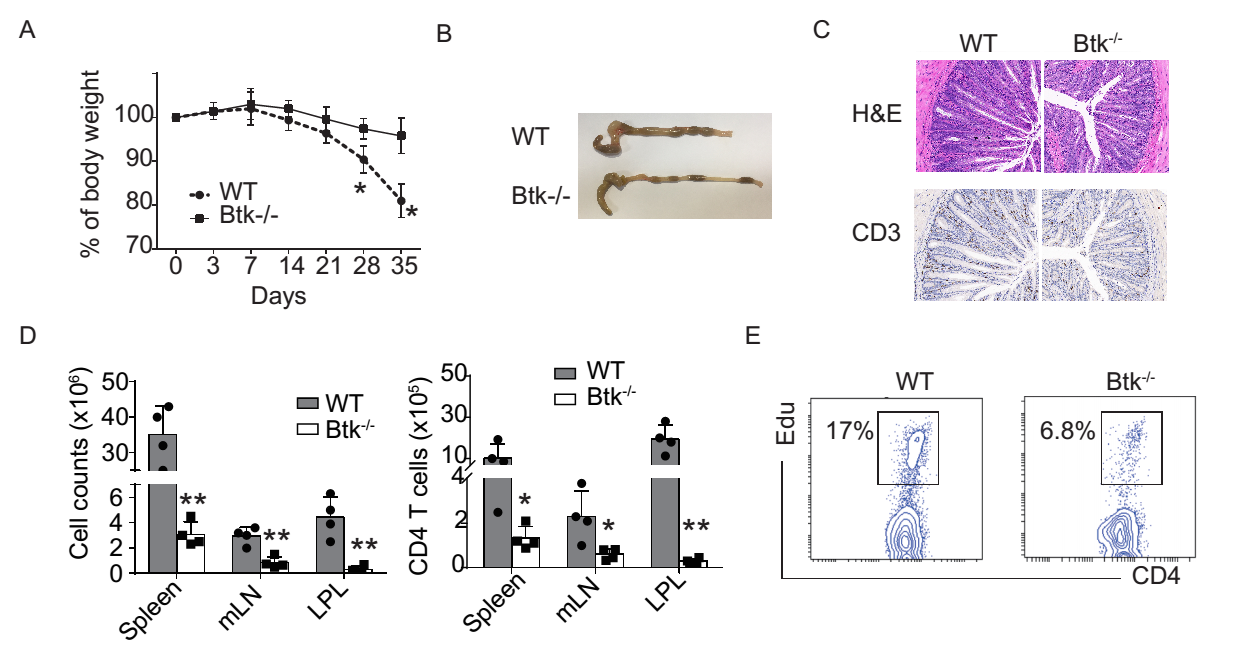
**Supplementary Figure 4. Btk deficiency ameliorate T cell mediated colitis**

(**A**) Body weight loss of Rag2^-/-^ mice adoptive transferred with WT or Btk^-/-^ naïve CD4^+^ T cells at indicated time points (n=5); (**B**) Representative plot of the colon from Rag2^-/-^ mice 5 weeks post transfer with WT or Btk^-/-^ CD4^+^ T cells; (**C**) Representative H&E staining and immunohistochemistry staining (anti-CD3) of the colon in (B), 400×; (**D**) Total cell count and CD4^+^ T cells of indicated organ from colitis mice; (**E**) Representative flow cytometry plots of Edu inoculation of CD4^+^ T cells from colitis mice. Data represent the mean ± SD or representative from 3 independent experiments.


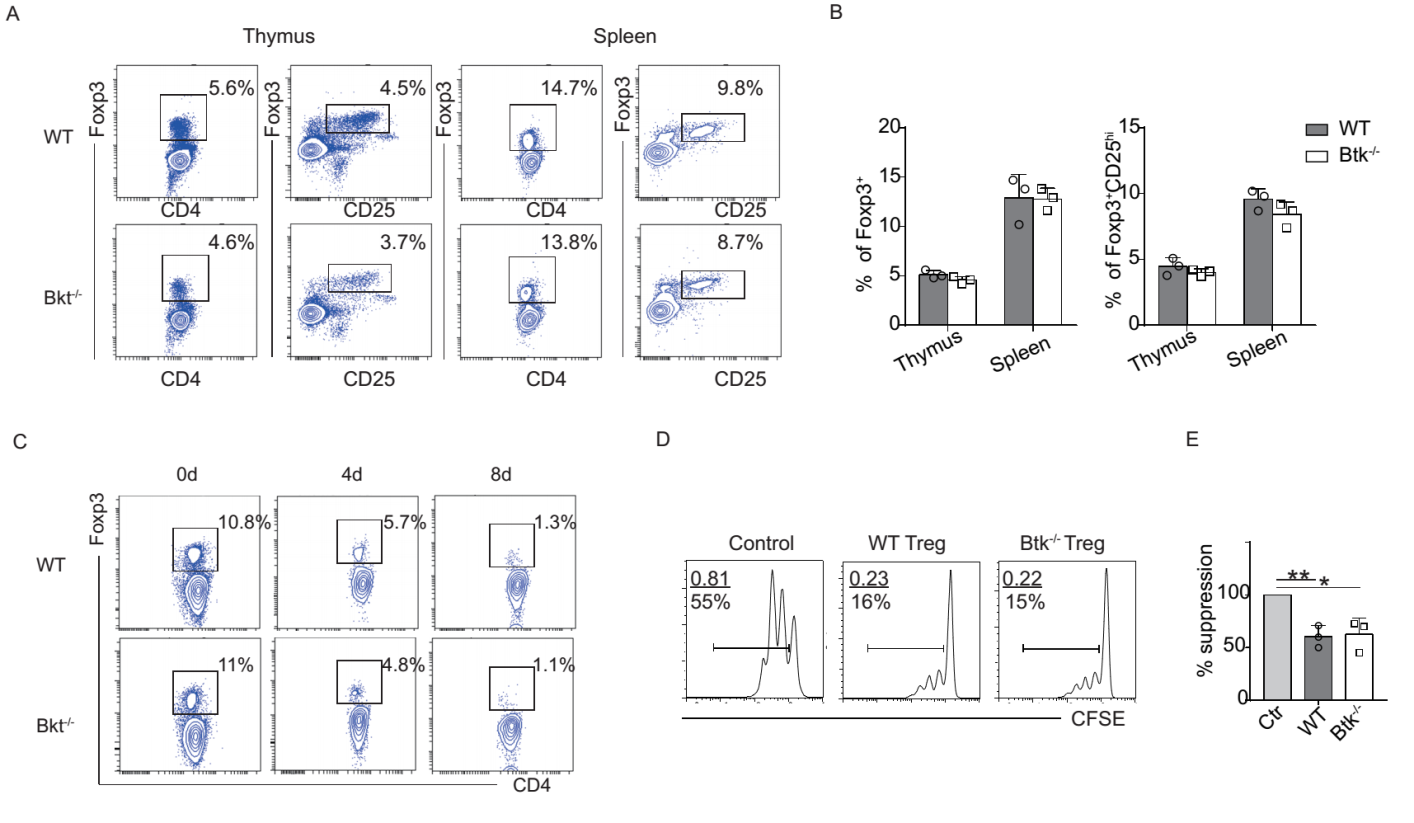


**Supplementary Figure 5. Btk deficiency did not affect Treg cell development and function**

(**A**) Representative flow cytometry plot shown Treg development in the thymus and spleen from WT and Btk^-/-^ mice; (**B**) percentages of Treg cells in WT and Btk^-/-^ mice; (**C**) Representative flow cytometry plot of Treg cells in the spleen from AA mice at indicated time post transfer with WT or Btk^-/-^ T cells; (**D**) Flow cytometry plots shown inhibition of CD45.1^+^ WT T cell proliferation by WT and Btk^-/-^ Treg cells; Number shown are divided index (upper) and % of proliferated cells (bottom) (**E**) Percentages of WT T cell proliferation suppressed by WT or Btk^-/-^ Treg cells. Data represent the mean ± SD or representative from 3 independent experiments.


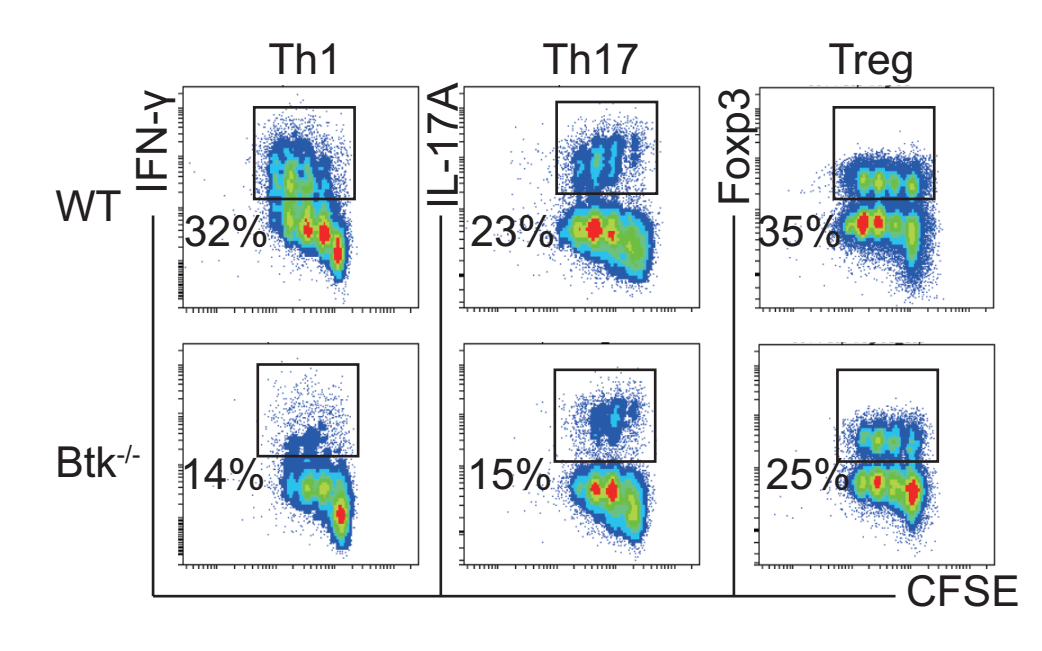


**Supplementary Figure 6. Helper T cell development of WT and Btk^-/-^ T cells**

Representative flow cytometry plots shown high cytokine production in CFSE low cells. WT and Btk^-/-^ CD4^+^ T cells were labeled with CFSE and then cultured under Th1, Th17 or iTreg conditions. Intracellular cytokine staining was done after 6h restimulation with PMA/ionomycin plus BFA. Data are representative from 3 independent experiments.


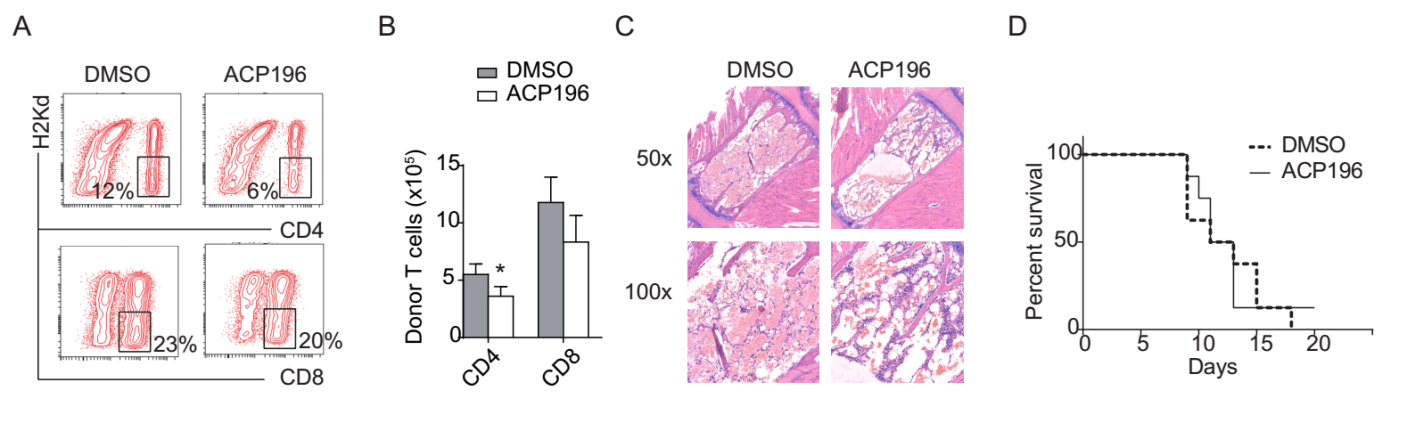


**Supplementary Figure 7 ACP196 slightly ameliorates mouse aplastic anemia after the onset of the disease**

(**A.B**) AA mice were treated with ACP196 or vehicle control 5 days post disease onset, the infiltration of donor T cells were analyzed on day 10 in the spleen (**C**), Representative hematoxylin and eosin staining of BM (**E**, upper 50×; bottom 100×); (**D**) Kaplan–Meier survival estimates for AA mice treated with ACP196 or vehicle control from day 5 (n=8). Data represent the mean ± SD or representative from 3 independent experiments. *, P < 0.05.
